# Novel Techniques to Improve Precise Cell Injection

**DOI:** 10.3390/ijms22126367

**Published:** 2021-06-14

**Authors:** Walter Linzenbold, Andreas Fech, Manuela Hofmann, Wilhelm K. Aicher, Markus D. Enderle

**Affiliations:** 1 Erbe Elektromedizin GmbH, 72072 Tuebingen, Germany; andreas.fech@erbe-med.com (A.F.); manuela.hofmann@erbe-med.com (M.H.); Markus.Enderle@erbe-med.com (M.D.E.); 2 Center of Medical Research, Department of Urology at UKT, Eberhard-Karls-University, 72074 Tuebingen, Germany; aicher@uni-tuebingen.de

**Keywords:** cell therapy, tissue engineering, regenerative medicine, medical technology, stress urinary incontinence, water-jet technology

## Abstract

We noted recently that the injection of cells with a needle through a cystoscope in the urethral sphincter muscle of pigs failed to deposit them nearby or at the intended target position in about 50% of all animals investigated (*n* > 100). Increasing the chance for precise cell injection by shotgun approaches employing several circumferential injections into the sphincter muscle bears the risk of tissue injury. In this study, we developed and tested a novel needle-free technique to precisely inject cells in the urethral sphincter tissue, or other tissues, using a water-jet system. This system was designed to fit in the working channels of endoscopes and cystoscopes, allowing a wide range of minimally invasive applications. We analyze key features, including the physical parameters of the injector design, pressure ranges applicable for tissue penetration and cell injections and biochemical parameters, such as different compositions of injection media. Our results present settings that enable the high viability of cells post-injection. Lastly, the method is suitable to inject cells in the superficial tissue layer and in deeper layers, required when the submucosa or the sphincter muscle of the urethra is targeted.

## 1. Introduction

In the last decades, the use of a water jet for surgical resection of human tissue (hydro-jet dissection) has been introduced and established as a dissection tool in many medical disciplines such as orthopedic surgery, neurosurgery, dermatology or urology [1,2,3,4,5]. Currently, hydro-jet technology is most frequently used to dissect the parenchymal tissue during partial hepatectomy or partial nephrectomy [6,7,8,9,10]. Hydro-jet dissection relies on an extremely thin, high-pressure water stream that can be used for penetration and selective separation of the tissue targeted. The selection is based on the stiffness and/or composition of the tissue. The hydro-jet delivers kinetic energy, and the cutting effect results in a mechanical fragmentation of the tissue [11]. By varying the pressure, the velocity or the probe parameters such as nozzle diameter, selective dissection and cutting of tissues of various consistencies and elasticities is feasible. Depending on the energy of the hydro-jet, tissues with low tensile strength and density, such as parenchymal tissue, are dissected, while others, such as bile ducts, blood vessels and nerves, remain intact [7,12]. Thus, bleeding and leakage, for example, from an incised renal or hepatic duct system is avoided [3,7,13]. Other advantages of hydro-jet dissection over thermal technologies (such as RF-surgery) are the absence of heat generation, which might cause thermal damage to the targeted tissue and surrounding area, and a better view of the surgical site [9,10,14,15,16]. This water-based technique has also been employed for endoscopic submucosal dissection (ESD) [17,18,19], peroral endoscopic myotomy (POEM) [20,21,22], Barrett’s esophagus [23] or submucosal tunneling endoscopic resection (STER) [24,25]. For such procedures, the water-jet delivers isotonic liquids in the targeted tissue without significant efflux of salts [26,27,28]; thereby, facilitating complex surgeries with a lower risk of unintended injury [26,29,30]. Based on these findings, we developed a novel technology for needle-free cell injections by water-jet.

Currently, the gold standard of cell delivery is the injection via a syringe and sharp hollow needle in veins [30,31]. For cell injections in tissue, nearly the same needles are used without significant modification. The sharp end of the cannula cuts through the tissue layers until the point of interest is reached. Then, the cells are administered. Even though single-needle injection is a widespread cell delivery technique, needle injections generally bear various disadvantages that may influence the viability, placement, retention rate or distribution of administered/injected cells [32,33,34]. Among these, the mechanical forces that cells may experience during a passage through the needle have been suggested to contribute to cell damage during delivery. Additionally, injection by cannulas causes trauma to the tissue in the range of the outer diameter of the cannula itself and causes the reflux of the cells along the injection channel when pulling out the injection needle [35]. To overcome this vicious circle, physicians tend to utilize thin needles to reduce tissue damage, bleeding and pain. In some cases, multiple injections per treatment session are applied to increase the distribution area of the therapeutic cells [32,33]. However, cell delivery by narrow needles translates in higher shear stress which negatively interferes with the viability of the cells [35]. In contrast, needle-free cell delivery by water-jet offers the possibility to deliver cells with high precision to the target region without causing “needle-stick” trauma. 

Jet injectors normally consist of a power source, some type of reservoir for the drug, a nozzle ranging in diameter from 76 to 360 µm, an energy system that is mediating force and mechanical elements such as a trigger [36]. Needle-free jet injectors have been used for vaccination as well as insulin delivery for years [36]. Recently, jet injectors are investigated for DNA delivery in tumor therapy [37]. Here we report on a novel technology replacing injection needles by water-jet to deliver viable cells on and in tissues.

## 2. Results

### 2.1. Design of the Injection System and Working Principle 

**Figure 1:**
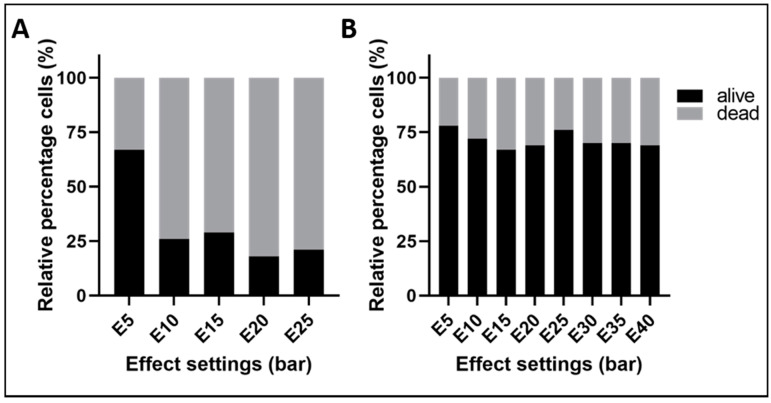
The relative percentage of living versus dead cells after injection at different pressures through a (**A**) narrow tube (caliber 120 μm, with nozzle) and (**B**) wide tube (caliber 500 μm, no nozzle). In very thin tubes with nozzle (**A**) at pressures above E5, 70–80% of cells are dead. In wider tubes (**B**) at pressures between E5 and E40, about 70–80% of cells were viable after water-jet injection. The figure shows the mean values of the representative experiment.

In the first set of experiments, straight tubes with or without a nozzle were employed to investigate the basic physical parameters of water-jet injections of cells (Figure 1). In these studies, established cell lines such as MonoMac6, HeLa, human umbilical vein endothelial cells (HUVEC) and human bone marrow-derived mesenchymal stromal cells (MSC) were used. The mean sizes of the cells in suspension employed here ranged from 13 μm (e.g., MonoMac6) to 22 μm (e.g., MSCs in suspension). Cell densities tested ranged from 10^4^ to 3 × 10^6^ cells per milliliter. Tube calibers varied from 100 μm to 500 μm, and pressure levels applied were effect E5 to E80 equivalent to approximately 5–80 bars. Please note that the term “effect” (abbreviated as “E” in this text and figures) is used in the context of this paper to refer to the pressure applied by the water-jet pumps. Using narrow tubes with a nozzle and effects at or above 10 bars reduced the percentage of viable cells after injection to or below 25% (Figure 1A). In contrast, the proportion of viable cells after injection was about 75% when tubes with a wider bore and no nozzle were used (Figure 1B).

**Figure 2:**
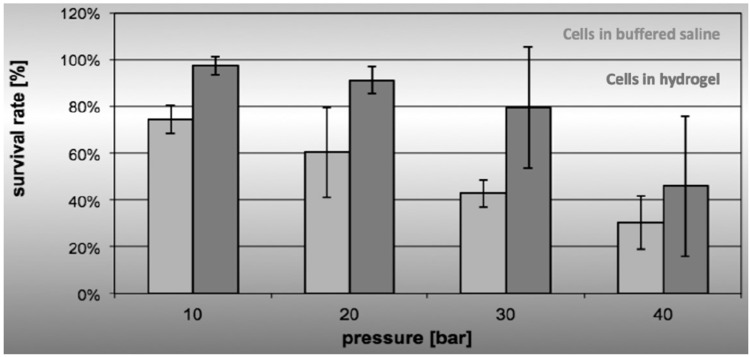
Cell viability after water-jet injection in isotonic solvents. Cells were in suspension in buffered saline (PBS, light grey bars) or in an isotonic hydrogel (PBS enriched by gelatin, dark grey bars) and injected in capture fluid applying different effects of E10 to E40 as indicated (x-axis). Cells injected in PBS enriched by gelatin yielded a higher normalized mean viability (y-axis, survival rate in %) when compared to cells in PBS only. Data presented show mean values ± standard deviations of *n* ≥ 6 individual measurements, using HeLa cells.

When investigating cell viability after water-jet injection in regards to the caliber and effect, we noted that the addition of proteins yielded more viable cells at a given effect or pressure when compared to water-jet injections of cells in buffered saline only (Figure 2). In this set of experiments, we compared the transportation media phosphate-buffered saline (PBS) with divalent Ca^++^ and Mg^++^ ions versus cell culture media (DMEM). Complementation of transportation media with type I collagen was halted as collagen tended to block narrow pipes even in comparably low–moderate concentrations. In contrast, the addition of gelatin facilitated cell injections at higher effects with sufficient viability (Figure 2).

**Figure 3:**
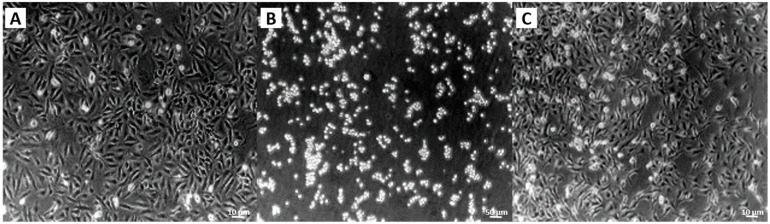
The effect of gelatin on the attachment of cells to culture vessels. Cells were re-suspended in cell culture medium and either seeded directly in cell culture vessels (**A**, left), or mixed 1:1 (**B**, middle), or 1:4 (**C**, right) with a gelatin stock solution, and then seeded. Cells seeded in a medium with a higher gelatin content failed to attach (**B**). They underwent cell death overnight, while cells seeded in medium without gelatin (**A**) or cells seeded in the presence of diluted gelatin (**C**) attached well and proliferated. The micrographs show a representative experiment using MSCs.

**Figure 4:**
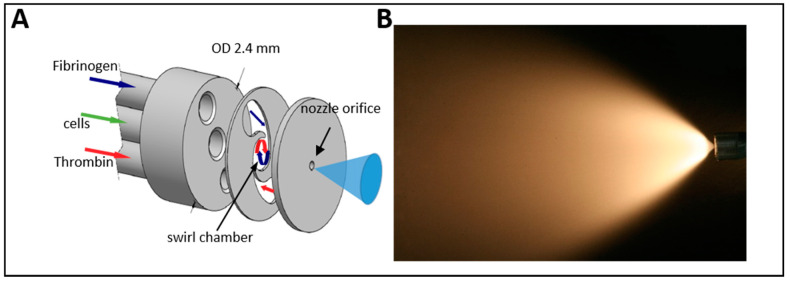
The injection/spray applicator for endoscopic use. (**A**) Design of the multi-channel prototype lance and working principle of a swirl-chamber mixing nozzle. (**B**) Wide-field spray-pattern of a jet generated with cells in transportation medium complemented by protein. This is the jet optimized for wide-field applications on surfaces.

However, the gelatin concentrations required to protect cells during water-jet injections inherited a significant disadvantage: Gelatin binds to integrin receptors on cells and inhibits the attachment of cells to the culture vessels (Figure 3). Integrin signaling is important for the cell survival of sessile cells such as mesenchymal stromal cells (MSC), chondrocytes, osteoblasts, smooth muscle cells and many others. Gelatin-coated cells remained in suspension and went into apoptosis overnight (Figure 3). We hypothesized that the same could occur after injection of cells in media enriched by high concentrations of gelatin after in vivo applications. In real-life situations, significant cell loss after injection (by apoptosis, for example) is not tolerated. In general, this study was designed to grant the final overall viability of cells at least at or above 80%. We, therefore, searched for other cell-protective proteins and explored three sets of self-polymerizing blends: (i) albumin plus a chemical crosslinking agent, (ii) gelatin plus transaminase and (iii) thrombin plus fibrinogen. The components were diluted in a way to allow injection in a liquid phase followed by polymerization within less than 2–3 min after water-jet injection. This required a novel design of the injector with separate tubes for the different components: (a) the cells resuspended in the protectant protein solution, (b) a scaffold material for polymerization and c) the corresponding catalyzer (Figure 4). 

**Figure 5:**
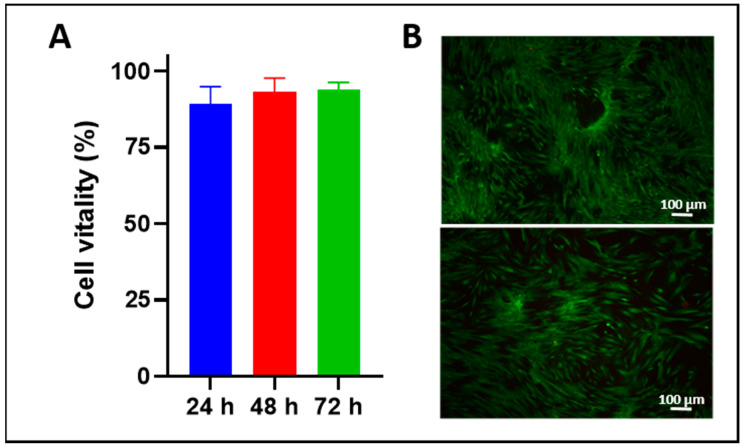
(**A**) The viability of cells 24, 48 and 72 h after injection. Calcein-labeled MSC were resuspended in DMEM medium complemented by 10% FBS, and a fibrin scaffold was generated by injection of fibrinogen plus thrombin from the lateral channels as well. Cell viability was determined in the scaffold after culturing the MSCs for the time indicated. Data present the mean ± standard deviation of *n* = 5 measurements. (**B**) Example of cells after 72 h of culture in fibrin scaffold. Viable cells emit green fluorescence when stained with calcein AM.

Based on this preliminary work, the first prototype for the injection of multiple components for gastrointestinal surgery was developed (Figure 4A). It consists of a thin straight pipe equipped with three different channels: a central channel for the delivery of a cell suspension and two lateral channels for the injection of supplements. Employing this design, three individual components can be transported separately through the instrument to the nozzle, mixed in the nozzle and injected into the target tissue (Figure 4A). Different combinations of active components were tested, including the synchronous injection of cells in medium, fibrinogen and thrombin from channels a), b) and c), respectively. The best fluidics and cell viability after water-jet injection were achieved by resuspension of cells in complete media enriched by 10% serum, in combination with fibrinogen and thrombin and at ambient temperature (data not shown). By adjusting the concentrations of fibrinogen and thrombin, the components polymerized within a few seconds after injection and generated a biocompatible hydrogel containing viable cells. As the scaffold material (here fibrinogen) and the catalyst (here thrombin) can be separately diluted in the reservoirs, different elasticities and densities of scaffolds for better cell nesting can be achieved. This allows adjusting the physical and biochemical characteristics of the implant injected to the tissue targeted and the regenerative need as well. Fibrin scaffolds with several millimeters of thickness containing viable cells were generated and cultured. The cells survived in the injected fibrin scaffold without any restriction at construct heights of up to 4 mm, and with high viability. Red fluorescence indicating nuclei of dead cells was rarely detected (Figure 5). 

The injection of solutions without cells and of micro- and nanoparticles was performed as well [38,39]. For sealing of wounds or related applications, the injection system also allows generating only scaffolds without delivery of active components such as cells or drugs. Defined gradients of up to three different components can be applied because each channel is equipped with an individual control unit, pump and reservoir.

In order to facilitate wide field injections (spraying), an optimized, semi-flexible prototype of a cone-shaped jet was introduced (Figure 4A). To this end, the fibrin glue substrates fibrinogen and thrombin are directed through the two lateral channels to the orifice of the instrument and are vaporized by a so-called “swirl pressure spray” nozzle (Figure 4A). Thrombin and fibrinogen enter the swirl chamber through narrow, tangential inlet channels resulting in a rotational flow within the swirl chamber (Figure 4A). There are two effects of the rotational flow: first, a thorough mixing of the components, and second, due to momentum conservation, the breakup of the jet into individual droplets that occur after the jet exiting the nozzle achieving the desired cone shape spray pattern (Figure 4B). Cells, on the contrary, are gently added into the swirl chamber through the central inlet channel reducing the mechanical stress to the cells. Subsequently, cells are incorporated into the rotating flow in the swirl chamber and carried out alongside the fibrinogen–thrombin mixture. Due to the very short contact time, no agglutination of the fibrin glue occurs during the active spraying process (Figure 4B).

**Figure 6:**
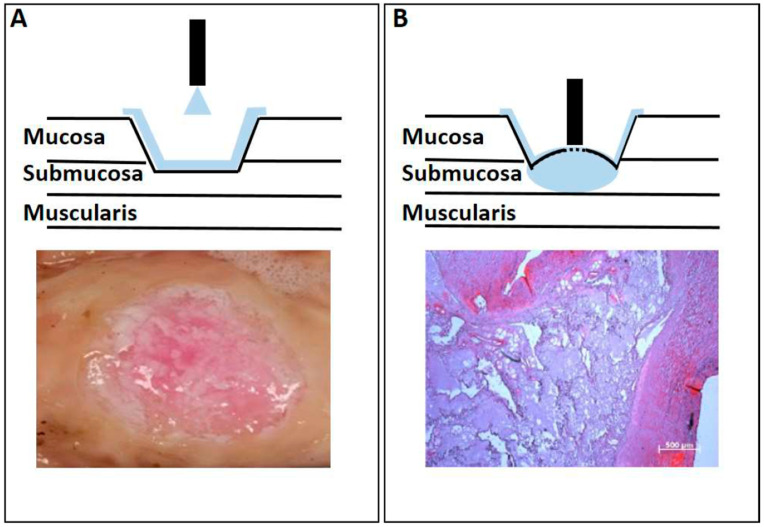
Ex vivo substrate deposition with spray applicator (**A**) Sealing a gastrointestinal wound by water-jet injection of a superficial layer on a gastric defect. The principle of the application is shown in the upper panel. The lower panel shows the aspect during a pre-clinical study. (**B**) Submucosal injection by water-jet for protection or treatment of the tissue. The principle of the application is shown in the upper panel. The lower panel shows an example of histological analyses of injections in deeper tissue layers (paraffin section).

The water-jet system with a cone-like spray function is a versatile tool. It can be used to superficially apply the fibrinogen–thrombin mixture on the lesion surface (Figure 6A) or to inject the mixture into the remaining submucosal layer (Figure 6B). To generate a superficial layer, the applicator is moved above the tissue at a distance of about 10–20 mm, leading to a homogenous layer on the tissue surface under visual control, e.g., by an endoscope. The thickness of the generated “protection shield” can be adjusted either by the speed of the movement or by the wiping number of the instrument over the target tissue. For submucosal injection, the nozzle of the instrument is touching—but not puncturing—the surface layer. Due to the low mechanical strength of submucosal tissue [40] and the high dynamic pressure at the exit of the nozzle, the liquid is injected into and widely distributed within the submucosal layer (Figure 6B).

### 2.2. Adapting the Water-Jet Technology to Urology for Transurethral Applications

**Figure 7:**
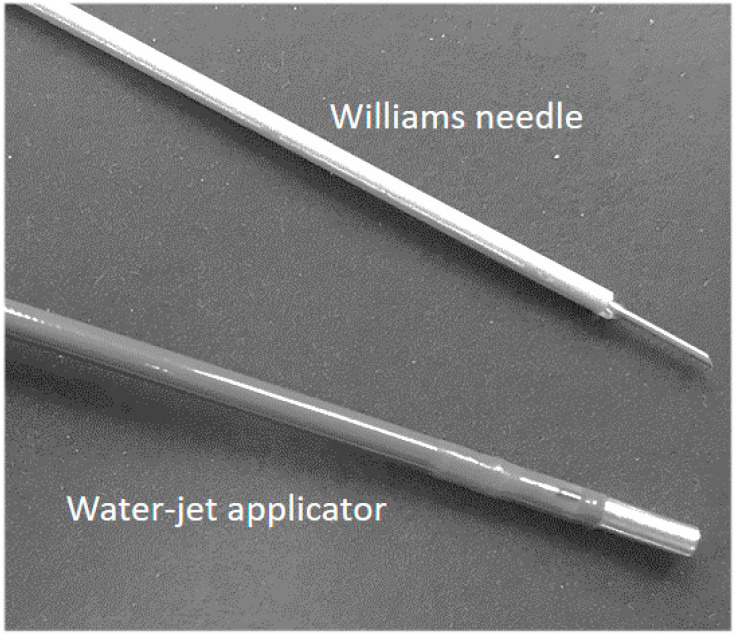
The prototype of a water-jet cell injector designed for use with a cystoscope. Cells can be injected into the urethral sphincter muscle by the cystoscope under visual control using a Williams needle (top). The water-jet injector used in this study is shown below.

Based on the injector developed for the application of cells and other active components in laparoscopic visceral surgery, an even smaller applicator was developed to fit the working channel of a standard cystoscope under visual control during the transurethral route. The outer diameter of this injector is 2.3 mm (Figure 7), matching the dimensions of a standard Williams needle used in clinical situations for injections of bulking agents in the urethra (Figure 7). However, in the female and even more in the male urethra, the angulation of the straight/rigid injector is limited. Therefore, a flexible, steerable injector with an angulation function was developed. Moreover, the design of tubing and nozzle had to be adapted again to facilitate penetration of cells through the urothelial cells and connective tissue in or next to the muscular layer of the urethra. In addition, pressure profiles for the injection of cells had to be adjusted as well [41,42].

Fresh porcine cadaveric sphincter samples were injected by water-jet with colored fluids or with fluorescent nano- and microparticles [38,39]. The particles employed had a density ρ of 1.05 mg/mL to mimic the mean density of nucleated cells. By these experiments, the injection depth in the tissue layer of the urethra could be studied. When applying pressures below E40, fluids and particles did not pass the urothelial layer but were found outside of the target tissue. When applying pressure above E80, full penetrations of the cadaveric urethra samples were observed, i.e., the urethra was perforated [38,39]. From these experiments, the applicable range of injection pressures could be derived.

**Figure 8:**
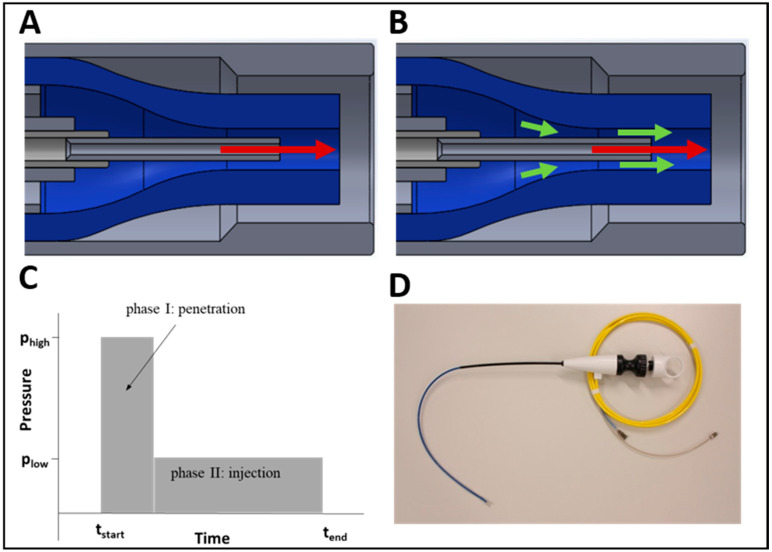
The instrument and phases for the injection of viable cells. PBS (red arrow in **A** and **B**) is injected at high pressure through a central nozzle in order to create an injection channel during phase I (**A**,**C**). Immediately after, the pressure of the PBS jet is instantly reduced (phase II in **C**) to a moderate pressure level, and cells (**B**) (green arrows) are gently added using a lower pressure to the jet exiting the central nozzle. The flexible instrument is displayed in (**D**).

**Figure 9:**
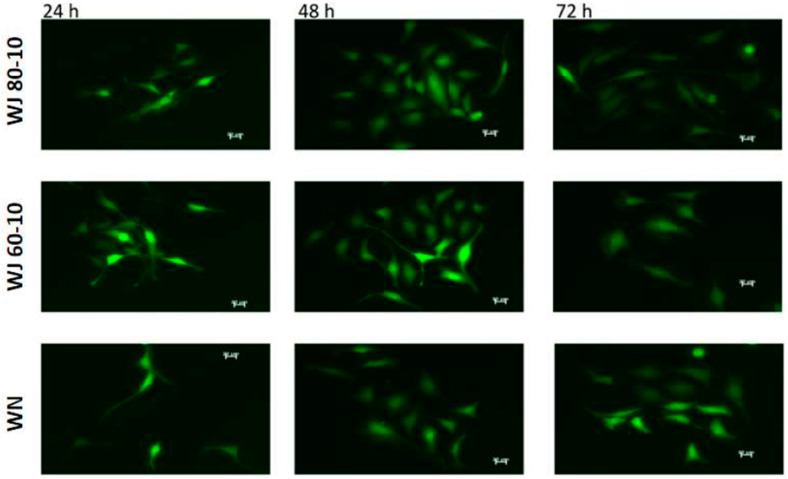
The injection of cells in porcine cadaveric tissue and cell retrieval. GFP-transduced HeLa cells were injected by water-jet using the E80-10 (top) or E60-10 (middle) injection mode in urethral sphincter tissue from fresh female cadavers. The cells were extracted and incubated for the time indicated. Cell injections by Williams needle (WN) served as controls. Differences in yield or viability were not seen during follow-up of 24–72 h. Size bars 20 μm.

However, at higher pressures and therefore at faster travel speed, cell viability decreases after injection through narrow tubes, and especially through pipes with nozzles (Figure 1 and Figure 2). For successful injection of viable cells in a tissue, therefore, an upgraded pump and controller system (UPaCS) operating at two distinct pressure levels and an improved pressure control mode (IPCM) were developed (Figure 8 and Figure 9A) [41,42,43]. This system allows cell administration into the sphincter muscular layer by a two-phase injection (Figure 8C).

In the first phase, a high-pressure jet of a pure PBS or other transport medium is applied at a pressure between 60 and 80 bars in order to loosen the extracellular matrix of the tissue on its way to the point of treatment and to open small interconnecting micro-lacunae for the cells next or within the muscle (phase I in Figure 8C).

The high-pressure water-jet is generated by a centrally aligned nozzle (inner diameter 0.12 mm; Figure 8A). In a second step, the pressure of the jet is instantly reduced to a moderate level (phase II in Figure 8C), and cells are gently added to the jet exiting the central nozzle (Figure 8B). Cells are introduced through a separate, annular channel concentrically aligned to the central high-pressure nozzle. The width of the annular channel is larger than the mean diameter of the typical cells, resulting in low mechanical stress. Cells are introduced into the central jet due to Venturi-effect. The jet is powered by a modified ERBEJET2 pump, allowing for a rapid and accurate change between higher pressure for tissue penetration and a lower pressure for cell injection pressures. An additional low-pressure pump is used to transfer the cells and provide accurate dosing of cell suspension.

By in vitro analyses, cell viabilities above 80% were observed upon injection of different somatic cells, including HeLa cells expressing recombinant green fluorescence protein (GFP), human and porcine MSC and porcine muscle-derived cells. Upon injection of such HeLa cells by water-jet in porcine cadaveric tissue samples, viable cells were isolated and expanded for several days (Figure 9). Significant differences in the yield of viable cells between water-jet vs. needle injections were not observed. This corroborated our recent studies [41].

**Figure 10:**
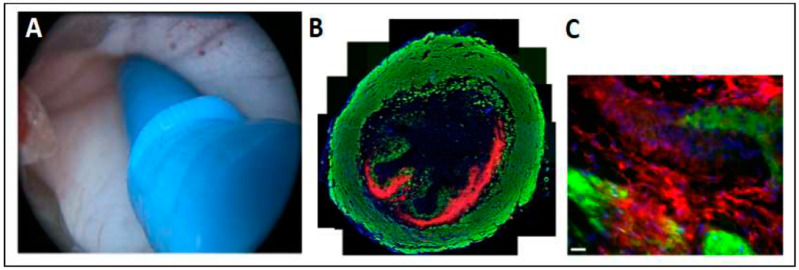
Endoscopic view during (**A**) cell injection by water jet injection. A wide cell distribution (red) was observed in cryosections near or in the muscle layer (green) in samples 3 days after water-jet injection (**B**,**C**). Size bar indicates 10 µm, magnification of image (**C**) was 20×. Figures modified with permission from [42].

We then went one step ahead and injected porcine adipose tissue-derived MSCs via a cystoscope under visual control into the sphincter complex of female living pigs using the transurethral route (Figure 10). Viable cells were observed in tissue samples after follow-up for up to 3 days [42].

Of note, after water-jet injection, cells were found in 90% of the urethra samples studies, whereas transurethral injection of cells by Williams needle in 45–70% were either not found at all in the urethra or were detected as misplaced [42,44]. Moreover, water-jet injections with the E60-10 protocol did not cause disruption of the muscle in any of the pigs (*n* = 26) and showed a much wider distribution of the cells compared to the conventional needle technique (Figure 10B,C) [42]. This technology and new technique have recently also been applied with great success in a preclinical animal study for cell injection in the porcine heart [45]. Other applications are currently under investigation.

## 3. Discussion

In one of our recent pre-clinical studies for cell therapy of stress urinary incontinence, more than 100 female Göttingen minipigs were included. This study provided evidence that nearly half of the animal cells injected by needle through a cystoscope did not reach the intended tissue layer [44]. Others corroborated this finding [46]. The proper delivery of regenerative cells is very critical to gain their optimal efficacy. Misplacement of cells may contribute to the very inconsistent outcome of clinical studies [47]. At the same time, minimally invasive surgical procedures require specific skills to inject active components close to the urethral sphincter muscle, which in adult women, for instance, is only a few millimeters wide and thick [48]. Therefore, several devices were designed to improve the precision of injection, albeit not always with satisfactory outcomes [49,50,51]. Extensive knowledge on selective and controlled tissue penetration by hydro-jet motivated us to explore the possibility to inject not only liquids enriched by hydrogels, dyes or macromolecules on or in tissues [19,27,28,52,53,54] but also deliver viable and regeneration-competent cells precisely in tissues [41,42]. Cells injected into the target tissue in situ support tissue regeneration for a short period. MSCs, for instance, release factors facilitating tissue regeneration and vascularization such as fibroblast growth factor (FGF) and vascular endothelial growth factor (VEGF), playing an important role in wound healing and neoangiogenesis facilitating better wound healing [55,56]. When cells are injected in combination with biomaterials, the biomaterial may even create a physical barrier avoiding loss of cells by efflux, entry, e.g., of urine or bacteria into the wound and prevents bleeding after injection. In gastrointestinal surgery, several studies have demonstrated that fibrin glue effectively prevents bleeding after ESD, avoids the entry of food into the wound, and plays a protective role for structure prevention [57,58,59]. The fibrin glue employed in our studies consisted of two components: (i) a solution of fibrinogen, factor XIII and phosphate buffer and (ii) a solution containing thrombin and CaCl_2_ turned out to be the preferred self-polymerizing biomaterial for the clinical application investigated here. It worked well with cells injected in media enriched by 5–20% FBS. It also generated in vitro porous, semi-solid scaffolds several millimeters high in which cells survived for several days. By variation of the concentrations of fibrinogen and thrombin, the stiffness of the scaffolds injected can be adapted to the stiffness of the tissue targeted or the clinical need. By variation of thrombin concentrations only, pore sizes may be varied as well. This, however, was not investigated in our experiments and must therefore await future studies. In addition, we hypothesize that for other clinical needs, chemically cross-linked gelatin or albumin may be better biomaterials than scaffolds generated by fibrin. Therefore, different combinations of biomaterials and regenerative cells must be explored depending on the clinical need.

The addition of biomaterials to the transportation buffer improved the yield and viability of cells injected by the water-jet technology. The same was observed for needle injections indicating that shear forces and contact of cells to the inner wall of the needle or tubing are critical parameters [31,60]. A recent study reported that only about 5% of the cell injected by needle persisted in tissues when no additional biomaterials were used [33]. When comparing cell injections by water-jet (viability above 85%) versus Williams needle (viability below 75%) in capture fluid, significant differences were noted [41]. However, after cell injections in cadaveric tissue samples by water-jet versus Williams needle, no significant differences in the yield of viable cells were found, confirming our recent study [41]. The apparent contradiction may be explained as follows: Changes in the diameters of the cell reservoir, transportation tubing, injection lance and nozzle of the water-jet system may cause elevated shear stress to the cells. This shear stress correlates to the travel velocity of cells [34]. However, due to the advanced pressure levels of the novel water-jet technology, this drawback can be avoided: Microchannels in the target tissue are opened with a short jet of PBS without cells at higher pressure. Within milliseconds, the system’s pressure is reduced to flush cells in suitable transportation at pressures at or below E10 in these microchannels. Thus, (i) shear stress is reduced by the low travel velocity of cells, and (ii) cells are not stressed when entering the tissue as micro cavernae are open. This, of course, is not the case to the same extent when cells are injected by needles. Here, cells “bumb” to a tissue, and the pressure applied generates a cavern, facilitating even reflux of cells when the needle is retracted [33]. We propose that the loss of cells after needle injection is associated with these two mechanisms: pressure against the tissue and reflux through the injection channel.

Last but not least, producing cells under GMP-compliant conditions and producing such cells, including autologous cells individually for each patient, is a time and cost-intensive business. The waste of precious cells by inferior cell injection technologies is therefore even more cumbersome. A metastudy recently compared the costs versus the outcome of cell therapy in the treatment of SUI in comparison to implantation of midurethral slings. The cell therapy was more effective, less invasive but more expensive [61]. Replacing needle injections by water-jet application of cells may reduce the costs for cell production when fewer cells are injected more precisely, avoiding loss of cells by reflux or full penetration [42]. The fewer cells injected by water-jet close to the sphincter muscle may even yield a comparable or even better outcome when compared to multiple injections of more cells by needle in one session, increasing the risk of injury considerably [49,62] or in repeated sections over time, which raises other concerns [63].

## 4. Materials and Methods

### 4.1. Waterjet Technology

The water-jet system includes a prototype of a modified water-jet device based on an ERBEJET 2 (Erbe Elektromedizin GmbH, Tuebingen Germany), the corresponding controller prototypes, and the injection lance itself. The system allows generating different pressures (1–80 bar) as well as different injection volumes starting at 50 μL. Through a software program, up to three devices can be connected and controlled simultaneously in order to transport different liquids with individual settings (i.e., pressure, volume) through multi-channel injectors (Figure 4 and Figure 8). For preparatory tests, open straight tubes with inner calibers ranging from 100 to 500 μm were employed as injection lances. To mimic the effects of nozzles, the orifice of the pipe was reduced by soldering metal semi-circles of different sizes on the tip. For gastrointestinal cell injections, a straight injection lance with three inner tubings and a mixing nozzle was developed (Figure 4). The three channels could be loaded with individual probes and liquids and injected by individual pumps. For transurethral injections, an advanced, smaller injection lance was developed, facilitating operation through a cystoscope (Figure 10). This device provided two channels for cells and other components, as was described, for example, in EP 3714926 A1 or EP 3040036 B1.

### 4.2. Production of Cells for Injections

HeLa cells (CRM-CCL-2, ATCC), MonoMac6 cells (ACC 124, DSMZ), NIH/3T3 fibroblasts (CRL.1658, ATCC) and pooled human umbilical vein endothelial cells (HUVECs; Lonza Biosciences, Basel, Switzerland) were obtained from providers and produced as requested in the manuals. Human bone marrow-derived mesenchymal stromal cells (MSCs) were obtained after informed and written consent (*n* = 12 donors), isolated from surgical surplus materials and produced as described recently [64]. The study was approved by the local Ethics Committee under file numbers 435/2011/BCO2 and 623/2013BCO2.

For the experiments, cells were washed twice with PBS. Adherent cells were detached from flasks by trypsin/EDTA (Thermofisher, Waltham, MA, USA), washed again in the corresponding cell culture medium and counted [64]. For injection experiments, cells were resuspended in PBS or in DMEM low glucose medium (Thermofisher, Waltham, MA, USA). To test biomaterials for improved cell injections, including higher yield and viability, PBS or DMEM were complemented either by gelatine (stock 0.1% in PBS; diluted 1:2 to 1:16, Sigma-Aldrich, Darmstadt, Germany), gelatine plus transaminase (Gelita, Eberbach, Germany), fibrin glue (diluted 1:4 to 1:10 in solvent; TISSEEL Fibrin Sealant, a generous gift from Baxter, Vienna; Austria, bovine albumin with a crosslinker (available from Cellendes upon request) or bovine serum (5–20%; Biochrom or Sigma-Aldrich, Darmstadt, Germany) at different concentrations. Cell viabilities before versus after injections were determined by cell counting with the aid of a hemocytometer and trypan blue dye exclusion, and in some experiments, also by the CASY cell counter (OMNI Life Science, Bremen, Germany). Cell sizes were determined by the CASY cell counter and by microscopy (C1 Observer, Zeiss, Oberkochern, Germany). The mean sizes of the cells in suspension employed here ranged from 13 μm (e.g., MonoMac6) to 22 μm (e.g., MSCs in suspension).

### 4.3. Labeling Cells

To detect cells after injection in biomaterials or to discriminate living injected cells from debris or host cells after injection in tissue samples, cells were labeled by calcein-AM and ethidium homodimer according to the manufacturer’s protocol (life/dead labeling kit, Thermofisher). Intact living cells appeared in green while nuclei of dead cells were red. Labelled cells were recorded by an inverse microscope (Axiovert A1; Zeiss, Oberkochern, Germany). For injection of cells in living animals, cells were labelled by PKH26 as requested by the supplier (Thermofisher).

### 4.4. Cell Injections

For injection experiments by needle or water-jet, cells were resuspended in a transportation medium as described recently [41,42]. In a first proof-of-principle study, the cells were, as described above, resuspended in different transportation media at different dilutions (1 × 10^4^ to 3 × 10^6^ mL^−1^). They were injected with pressures ranging from E5 to E80, corresponding to 5–80 bar in injection volumes starting at 50 μL. In the first set of experiments, where cell viabilities were tested in vitro, cells were injected in 50 mL centrifugation tubes filled with 10 mL of the corresponding media. The injection temperature of the transportation media investigated was 20 °C. The yield and viability of cells after injection was determined by cell counting using the trypan blue method and hemocytometer or the CASY counter. In the cadaver or living animal studies, cell injections were performed with the advanced lance as described recently [41,42]. In brief, the localization of the sphincter muscle in the urethra was determined by transurethral urodynamics in each pig prior to water-jet injection [44]. Labeled cells were loaded in the reservoir of the water-jet system, and the hose and tubings were filled with the cell suspension by repeated short pump cycles. Then, the desired injection mode E60 or E80 was selected, and cells were injected into the sphincter complex of the pigs [41,42]. The animal study was approved by the local Animal Welfare Authorities at Tuebingen, Germany under file number CU1/16; NTP9547-1-3.

### 4.5. Histology

To detect cells after injection in animals, pigs were sacrificed, and the urethrae were prepared as described in [42]. The region of interest was localized by an in vivo imaging system (IVIS; Perkin Elmer, Hamburg, Germany), and cryosections were generated from the corresponding part of the urethra. To visualize cell nuclei, cryosamples were stained by DAPI. To visualize muscular tissue, samples were incubated in a solution containing phalloidid-iFluor488 (1:1000; AAT Bioquest, Sunnyvale, CA, USA). Somata of injected PKH26-labelled cells appeared red in fluorescence microscopy and were recorded using a laser scanning microscope equipped with optical sectioning and a motorized sample table facilitating automated 3D scans of complete cryosections (C1 observer, apotome, LSM510 meta, Zeiss, Oberkochern, Germay). Micrographs were processed and mounted using the proprietary software programs (Zen blue, Zen black, Zeiss, Oberkochern, Germany).

### 4.6. Statistics

Data were processed by spreadsheet program (MS Excel) and imported to a statistics program GraphPad Prism v7.0 (GraphPad Software, San Diego, CA, USA). Mean values and statistical significance were computed. Chi-squared tests were used for contingencies. *p*-values below 0.05 were considered significant.

## 5. Conclusions

The novel water-jet technology facilitates a simple, fast and safe method to inject cells in delicate tissues such as gastrointestinal mucosa and submucosa or the urethra. The method employs minimally invasive instruments. The maximal reach of the jet can be limited to the injection depth required allowing a significantly low risk of cell loss due to misplacement, full penetration or even perforation. Cell viability was comparable to a needle injection technique. In future studies, injections of cells and other active components in other organs and tissues should be explored.

## Data Availability

Not applicable.
